# Pain perception and processing in individuals with posttraumatic stress disorder: a systematic review with meta-analysis

**DOI:** 10.1097/PR9.0000000000000849

**Published:** 2020-09-17

**Authors:** Jonas Tesarz, David Baumeister, Tonny Elmose Andersen, Henrik Bjarke Vaegter

**Affiliations:** a Department of General Internal Medicine and Psychosomatics, Medical Hospital, University of Heidelberg, Heidelberg, Germany; b Department of Psychology, University of Southern Denmark, Odense, Denmark; c Pain Research Group, Pain Center, Department of Anesthesiology and Intensive Care Medicine, University Hospital Odense, University Hospital Odense, Odense, Denmark; d Department of Clinical Research, Faculty of Health Sciences, University of Southern Denmark, Odense, Denmark

**Keywords:** Posttraumatic stress, PTSD, Somatosensory testing, QST, Systematic review, Meta-analysis, Combat, Trauma, Stress, Pain threshold, Pain tolerance, Pain sensitivity, Conditioned pain modulation, Wind-up

## Abstract

Posttraumatic stress disorder (PTSD) is a known risk factor for the development of chronic pain conditions, and almost 1 in 5 individuals with chronic pain fulfills the criteria for PTSD. However, the relationship between PTSD and pain is poorly understood and studies on pain perception in patients with PTSD show inconsistent results suggesting that different sensory profiles exist among individuals with PTSD. Here, we (1) systematically summarize the current literature on experimentally evoked pain perception in patients with PTSD compared to subjects without PTSD, and (2) assess whether the nature of the traumatic event is associated with different patterns in pain perception. The main outcome measures were pain threshold, pain tolerance, and pain intensity ratings as well as measures of temporal summation of pain and conditioned pain modulation. A systematic search of MEDLINE, EMBASE, Web of Science, PsycINFO, and CINAHL identified 21 studies for the meta-analysis, including 422 individuals with PTSD and 496 PTSD-free controls. No main effect of PTSD on any outcome measure was found. However, stratification according to the nature of trauma revealed significant differences of small to medium effect sizes. Combat-related PTSD was associated with increased pain thresholds, whereas accident-related PTSD was associated with decreased pain thresholds. No clear relationship between PTSD and experimentally evoked pain perception exists. The type of trauma may affect pain thresholds differently indicating the presence of different subgroups with qualitative differences in pain processing.

## Abstract 2

Supplemental Digital Content is Available in the Text.

Posttraumatic stress disorder encompasses latent subgroups of individuals with qualitative differences in pain perception.

## 1. Introduction

Posttraumatic stress disorder (PTSD) presents as a complex and protracted clinical picture with often severe social and health consequences for those affected. The symptoms of PTSD are very heterogeneous and varied, and in addition to the classic symptom triad of intrusion, avoidance, and hyperarousal, PTSD is characterized by a high comorbidity with chronic pain. Prevalence rates of chronic pain vary depending on how PTSD is assessed and defined, but self-reported pooled prevalence of PTSD is above 20% in individuals with chronic pain.^46^ Moreover, individuals with pain and comorbid PTSD report greater pain severity^5^ and pain-related impairment.^12^ Although there is strong evidence that PTSD is a risk factor for the development of chronic pain conditions, the underlying mechanisms are not well understood and may vary depending on trauma type.

The findings of previous studies examining pain perception in individuals with the presence of PTSD are inconsistent and partly contradictory, with results showing increased,^9,11,53^ decreased,^5,7,15^ as well as unaltered pain perception.^24,31^ On the one hand, these inconsistent observations could indicate that PTSD has no coherent influence on somatosensory pain measures. On the other hand, this ambiguity may be due to PTSD being a very heterogeneous, highly comorbid disease.^26,27,35,38,40,54^ Indeed, it has been suggested that PTSD may consist of latent subgroups of individuals with qualitative differences in the clinical manifestations.^4,17,23,30^ This seems to be especially true for the connection between trauma and pain because 2 important PTSD-related symptom clusters, anxiety and dissociation, are associated with opposing effects (eg, hyperalgesic vs hypoalgesic) on pain perception.^7^ Furthermore, there is evidence to suggest that interpersonal traumas are associated with higher PTSD risk and more complex trauma reactions compared to accident-related traumas, and may affect the sensory profiles in different ways.^25^

The differentiation and characterization of these subgroups is crucial for a better understanding towards the process of pain chronicity in individuals exposed to trauma.

A promising approach to explain contradictory data and to understand heterogeneity is the use of meta-analysis. Therefore, this article aims to (1) systematically review and meta-analyse current data on pain perception of experimentally evoked pain in individuals with PTSD compared to individuals without PTSD, and (2) compare whether the nature of the traumatic event is associated with different sensory profiles.

## 2. Methods

### 2.1. Procedures

This review was performed according to the recommendations of the Cochrane Collaboration^21^ when appropriate and is reported after the PRISMA statement.^36^ All steps and methods of the review were specified in advance in a predetermined review protocol registered with PROSPERO (CRD42018083779).

We searched Medline, EMBASE, Web of Science, PsycINFO, and CINAHL. The search strategy was adapted for each database if necessary (see the web appendix for complete search strategy, available at http://links.lww.com/PR9/A78). In addition, reference lists from identified articles and reviews were screened for published and unpublished data, and all promising references were scrutinized. For promising abstracts, complete publications were retrieved. In addition, a citation search on the included articles was performed. Searches were performed independently by 2 reviewers (J.T. and H.B.V.). Only peer-reviewed studies that were published in English, German, Danish, Swedish, or Norwegian were included. The 2 reviewers independently scanned the titles and abstracts of eligible studies. Both reviewers independently scanned the full-text articles to determine whether the articles met the selection criteria. Disagreements between the 2 reviewers were resolved by discussion, and if agreements between the 2 reviewers could not be achieved, a third reviewer was consulted (T.E.A.).

### 2.2. Eligibility criteria

We selected all studies that investigated measures of pain thresholds, pain tolerance, pain intensity ratings, temporal summation of pain, and conditioned pain modulation (CPM) in human adults older than 18 years of age with PTSD compared with a PTSD-free control group. In studies with more than one control group, the control group clinically closest to the PTSD group was chosen (eg, trauma-exposed controls without PTSD before healthy controls). The presence of possible PTSD had to have been qualified either (1) by interview according to either Diagnostic and Statistical Manual of Mental Disorders/DSM^1^ or *International Classification of Diseases*/*ICD*^55^ system or (2) according to a preestablished cutoff criteria for one of the existing validated PTSD questionnaires.

### 2.3. Outcomes

The primary outcomes were the more static measures of pain perception in experimentally evoked pain, namely pain thresholds, pain tolerance, and pain intensity ratings as well as the more dynamic measures of pain processing, namely temporal summation of pain and CPM. For this purpose, the following definitions were used.

#### 2.3.1. Pain threshold

Pain threshold was defined as the minimum intensity of a stimulus that was perceived as painful.^33^

#### 2.3.2. Pain tolerance

Pain tolerance was defined as the length of time an individual was willing to endure a noxious stimulus (eg, cold-pressor task) or by the maximum stimulus intensity that one was willing to endure in a given situation.^33^

#### 2.3.3. Pain intensity rating

Pain intensity rating was defined as the individual pain intensity rating (eg, on a 0–10 numerical rating scale) an individual perceived based on a given noxious stimulus (eg, pressure).

#### 2.3.4. Temporal summation of pain

Temporal summation of pain was defined as the amplification of pain intensity ratings after repeated or continuous administration of constant noxious stimuli (eg, pressure or heat). The presence of temporal summation of pain or wind-up indicates the involvement of sensitization processes at spinal neurons rather than damage or inflammation of peripheral structures.^32^

#### 2.3.5. Conditioned pain modulation

Conditioned pain modulation captures the phenomenon when the perception of a test-pain stimulus given together with (or directly after) a second painful conditioning stimulus is perceived differently than when the test stimulus is given alone. The extent of perceived pain reduction represents the value of CPM efficiency and has in the case of a perceived pain reduction usually a sign of “minus”; in the case when no pain decrease can be observed, the value of CPM is zero or positive and it is considered as less-efficient CPM.^56^

### 2.4. Assessment of risk of bias

A modification of the Newcastle–Ottawa Scale^19^ was used for judging the risk of bias of the included studies. The Newcastle–Ottawa Scale is a priori standardized checklist of predefined criteria for assessing the quality of nonrandomized cohort studies in meta-analyses. It assesses the quality of nonrandomized studies with its design, content, and ease of use directed to the task of incorporating the quality assessments in the interpretation of meta-analytic results. According to the Newcastle–Ottawa Scale, the quality scoring was divided into 3 sections: (1) Selection (including 3 items: representativeness of the cohort with PTSD, selection of the PTSD-free control cohort, and ascertainment of exposure/PTSD diagnosis); (2) Comparability (including 2 items: Comparability of cohorts by controlling for age and sex, and comparability of cohorts by controlling for pain at the test side); and (3) Outcome (including 2 items: Blinded assessment of outcomes, and assessment of outcomes in the same body area for both samples). An additional item considered obvious methodological flaws (eg, high dropout rate). Its content validity and interrater reliability have been established.^19^ Its criterion validity with comparisons to more comprehensive but cumbersome scales and its intrarater reliability are currently being examined. The checklist was slightly adapted to the specific needs of this systematic review. Before the registration of the final protocol, 3 articles were assessed and data extraction conducted by J. Tesarz and H.B. Vaegter to establish the consistency in the procedure and to adapt the data extraction form and the scoring system to the needs of this review.

### 2.5. Statistical analysis

Standardized mean differences (SMDs) were calculated as Hedges g for each measure of pain perception, comparing the individuals with PTSD with PTSD-free controls for each study. To compare and combine the different studies, we used a DerSimonian–Laird random-effects model^8^ to calculate pooled estimates with 95% confidence intervals (CI_95_). A random-effects model was chosen because studies differed in the nature of trauma and kind of pain induction methods.^8^ Heterogeneity among the studies was described using the I^2^ statistic, and I^2^ values over 50% indicated strong heterogeneity.^22^

Potential small study bias (ie, the association of publication probability with the statistical significance of study results) was investigated using Begg and Egger tests. All calculations were performed with the *metan* and *metabias* packages for STATA.

## 3. Results

### 3.1. Characteristics of included studies

Overall, 21 studies including 422 individuals with PTSD (201 women and 221 men) and 496 PTSD-free controls (267 women and 229 men) provided data for our meta-analysis (Figure S1 of the supplemental material, available at http://links.lww.com/PR9/A78). Seven of these 21 studies were conducted in the United States,^31,34,37,42,43,50,51^ 2 studies were conducted in Australia,^11,49^ 3 studies were performed in Israel,^5–7^ and 9 studies were performed in Europe.^9,13,15,20,24,28,29,45,53^ Veterans/victims of war/torture were assessed in 9 studies^5,6,13,29,31,42,43,50^; PTSDs that were closely linked to traffic accidents were assessed in 4 studies,^11,20,47,48,53^ and 4 studies assessed samples with “mixed” trauma types. In 4 studies, the type of trauma was not sufficiently described (see also Table S1 of the supplemental material, available at http://links.lww.com/PR9/A78). For the majority of studies, a low risk of bias was found (see Table S2 of the supplemental material, available at http://links.lww.com/PR9/A78).

### 3.2. Meta-analysis

#### 3.2.1. Pain threshold

**Figure 1.**
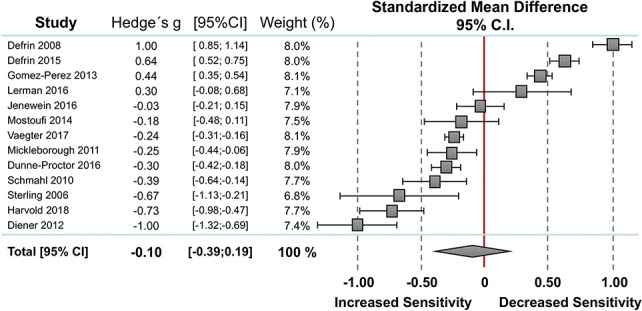
Overall effect on pain threshold. Standardized mean differences (SMDs) were calculated as Hedges g. A DerSimonian–Laird random-effects model was used to calculate pooled estimates with 95% confidence intervals (95% CI). Heterogeneity among the studies was I^2^ = 97.8%, *P* = 0.000.

Thirteen studies reported data on pain threshold in individuals with PTSD (n = 299) and without PTSD (n = 385). Random-effect analysis showed no significant difference between the 2 groups (Fig. 1). The pooled standardized mean difference was −0.10 with a CI_95_ −0.39 to 0.19 (I^2^ = 97.8%). Compared to controls, 7 studies showed lower pain thresholds in individuals with PTSD,^9,11,20,34,45,47,53^ 3 studies showed higher pain thresholds in individuals with PTSD,^5,7,15^ and 3 studies found no significant differences between the 2 groups.^24,31,37^ The funnel plot was symmetrical with no evidence of relevant small study bias (Egger test: *P* = 0.465).

#### 3.2.2. Pain tolerance

**Figure 2.**
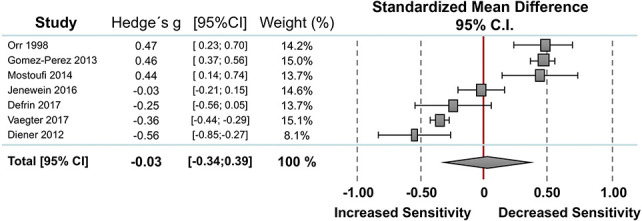
Overall effect on pain tolerance. Standardized mean differences (SMDs) were calculated as Hedges g. A DerSimonian–Laird random-effects model was used to calculate pooled estimates with 95% confidence intervals (95% CI). Heterogeneity among the studies was I^2^ = 97.2%, *P* = 0.000.

Seven studies reported measures on pain tolerance including 167 individuals with PTSD and 180 PTSD-free controls (Fig. 2). Random-effect analysis showed no significant difference between the 2 groups. The pooled standardized mean difference was 0.03 with a CI_95_ −0.34 to 0.39 (I^2^ = 97.2%). Compared to controls, 2 studies showed lower pain tolerances in individuals with PTSD,^9,53^ 3 studies showed higher pain threshold in PTSD,^15,37,42^ and 2 studies found no significant differences between the 2 group.^6,24^ The funnel plot was symmetrical with no evidence of relevant small study bias (Egger test: *P* = 0.708).

#### 3.2.3. Pain intensity ratings

**Figure 3.**
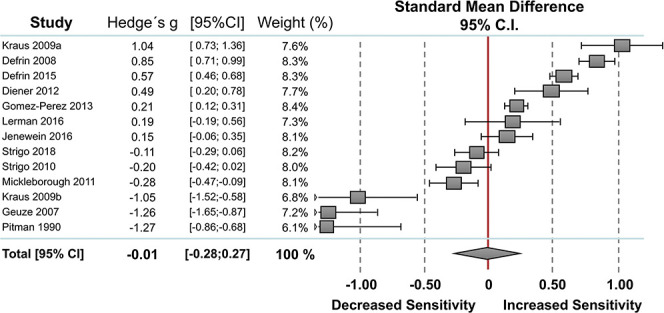
Overall effect on pain intensity. Standard mean differences (SMDs) were calculated as Hedges g. A DerSimonian–Laird random-effects model was used to calculate pooled estimates with 95% confidence intervals (95% CI). Heterogeneity among the studies was I^2^ = 96.1%, *P* = 0.000.

Thirteen studies reported measures of pain intensity ratings including 257 individuals with PTSD and 264 PTSD-free controls (Fig. 3). Random-effect analysis showed no significant difference between the 2 groups. The pooled standardized mean difference was −0.01 with a CI_95_ −0.28 to 0.27 (I^2^ = 96.1%). Compared to controls, 4 studies showed lower pain intensity ratings in individuals with PTSD,^13,29,34,43^ 5 studies showed higher pain intensity ratings in individuals with PTSD,^5,7,9,15,28^ and 4 studies revealed no significant differences between the 2 groups.^24,31,50,51^ The funnel plot was symmetrical with no evidence of relevant small study bias (Egger test: *P* = 0.094).

#### 3.2.4. Temporal summation

**Figure 4.**
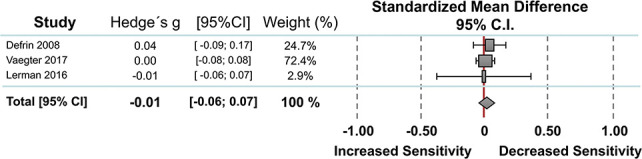
Overall effect on temporal summation of pain. Standardized mean differences (SMDs) were calculated as Hedges g. A DerSimonian–Laird random-effects model was used to calculate pooled estimates with 95% confidence intervals (95% CI). Heterogeneity among the studies was I^2^ = 0%, *P* = 0.000.

Three studies reported data on temporal summation of pain between individuals with PTSD (n = 86) and without PTSD (n = 91). Random-effect analysis showed no significant difference between the 2 groups (Fig. 4). The pooled standardized mean difference was 0.01 with a CI_95_ −0.06 to 0.07 (I^2^ = 0%, *P* = 0.886). The funnel plot was symmetrical with no evidence of relevant small study bias (Egger test: *P* = 0.873).

#### 3.2.5. Conditioned pain modulation

Two studies reported data on CPM between individuals with PTSD (n = 56) and without PTSD (n = 78). No meta-analysis was possible due to the small number of included studies. Vaegter et al.^53^ observed no differences in CPM comparing patients with accident-related back pain with PTSD to those without PTSD (Hedges g = −0.13 ± 0.04), whereas Defrin et al.^6^ observed reduced CPM in individuals with chronic combat-/torture-related PTSD and delayed-onset PTSD compared (Hedges g = −1.19 ± 0.18) to 2 PTSD-free groups (resilient and healthy controls).

#### 3.2.6. Subgroup analyses

**Figure 5.**
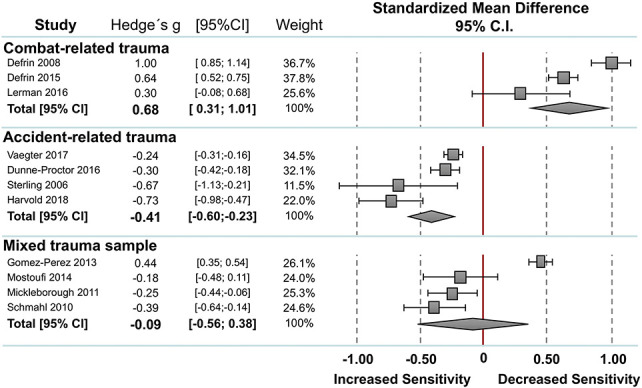
Stratification for kind of trauma for pain threshold measures. Standard mean differences (SMDs) were calculated as Hedges g. A DerSimonian–Laird random-effects model was used to calculate pooled estimates with 95% confidence intervals (95% CI). Heterogeneity among the studies was I^2^ = 90.4%, *P* = 0.000 for the combat-related trauma sample, I^2^ = 81.1%, *P* = 0.000 for the accident-related trauma sample, I^2^ = 89.8%, *P* = 0.000 for the sample without comorbid pain conditions, and I^2^ = 81.1%, *P* = 0.001 for the sample with comorbid pain conditions.

Stratifying pain threshold measures for the kind of trauma, subgroup analyses showed significant increased pain thresholds in subjects with PTSD with combat-related trauma in terms of a “hypoalgesic effect” of medium effect size (Hedges g = 0.68, CI_95_ 0.36–1.01; I^2^ = 90.4%; n = 3), and significant decreased pain thresholds in terms of a “hyperalgesic effect” of small effect size in subjects with PTSD with accident-related trauma (Hedges g = −0.41, CI_95_ −0.60 to −0.23; I^2^ = 81.1%; n = 4) (Table S3, available at http://links.lww.com/PR9/A78, Fig. 5). No significant difference was seen for the mixed-trauma group (Hedges g = −0.09, CI_95_ −0.56 to 0.38; I^2^ = 95.9%; n = 4). Due to the limited number of studies included, subgroup stratification was only possible for mixed trauma sample regarding the pain tolerance measures and for combat-related trauma regarding the pain intensity measures, indicating no significant differences within these subgroups. No subgroup analyses were possible due to the small number of included studies for the measures of temporal summation and CPM.

## 4. Discussion

### 4.1. Summary of findings

Based on a systematical summary of the current evidence on pain perception of experimentally evoked pain in individuals with PTSD compared with individuals without PTSD, no clear relationship between PTSD and pain perception exists. The type of trauma revealed some significant differences of small to medium effect sizes between individuals with PTSD and without PTSD, possibly suggesting that groups of individuals with PTSD consists of subgroups with qualitative pain perception differences. However, the available data are extremely heterogeneous, from small sample sizes, and limited to inclusion of only a few trauma types.

### 4.2. Subgroups stratified according to the nature of traumatization

With regard to the nature of traumatization, the analyses suggest that combat-related PTSD is associated with increased pain thresholds, whereas accident-related PTSD is associated with decreased pain thresholds. Alterations in pain thresholds seem to be related to the underlying trauma rather than to the presence of PTSD symptoms per se, and may indicate differential underlying mechanisms and studies comparing accident-related trauma with combat-related trauma are warranted.

One possible hypothesis behind a difference in pain threshold between trauma types is that complex PTSD symptomatology is more common in combat-related trauma compared to accident-related trauma and whiplash-associated disorder. This may affect coping mechanisms. For example, the dissociative subtype of PTSD is rarely seen in whiplash-associated disorder,^18^ whereas pronounced levels of dissociation have been seen in complex PTSD.^3^ Subjects with accident-related or whiplash-associated PTSD conditions may show more pronounced levels of anxiety and pain catastrophizing, and lower levels of dissociation.^18^ It has been shown that experimental pain ratings are negatively related to dissociation levels,^7^ and positively related to anxiety associated with fearful appraisals of pain.^41^ It has therefore been suggested that reduced conscious attention allocated toward incoming stimuli, resulting from dissociation, causes delayed response in pain threshold measurement, whereas biases toward threatening and higher intensity stimuli are responsible for the intensification of experimental and chronic pain.^7^ Posttraumatic stress disorder may thus lead to both habituation and sensitization according to this dual process theory—depending on whether one or the other process predominates, a hypoalgetic or a hyperalgetic effect occurs.

A similar phenomenon of psychophysiologically higher pain threshold on the one hand and increased risk for chronic pain on the other is known from studies with patients with borderline personality disorder, which is closely associated with early childhood trauma. This phenomenon was explained by a lower body perception^14^ and suppression of emotional experiences^10^ in those affected. It was postulated that this leads to an increased sympathetic activity and an increase in allostatic load, which in turn is a risk factor for the development of chronic pain in many ways.^10^ Others have suggested that decreased acute pain sensitivity is a result of stress-induced analgesia or dissociation,^2^ and alterations in the brain's regulation of the affective motivational component of pain.^39^ Increased chronic pain sensitivity has been conceptualized as a failure to self-regulate^44^ or has been linked to depression.^52^

An alternative hypothesis is related to the willingness to report pain. As pain endurance is a part of military training and practice, veterans might lack motivation to report pain (“stoicism”). In this regard, “stoic veterans” should feel as much pain as others but express their experience less. In the included studies, pain reports relied on subjective pain ratings and may therefore have given the appearance of increased pain thresholds. This idea is supported by the study of Kraus et al.,^29^ which reported similar pain thresholds between veterans with and without PTSD, but significantly higher pain thresholds in veterans compared to healthy controls. Yet, the data provided by Defrin et al.^5,7^ suggest paradoxical effects of both hyperalgesia and hypoalgesia in combat veterans, underlining that any conclusions about the latent causes of the apparent effect remain highly speculative and should be considered with caution.

A possible additional interference effect must also be taken into account here: the studies on accident-related trauma predominantly included patients with concomitant comorbid pain conditions (eg, whiplash), whereas studies on veterans or other traumas included heterogeneous or even pain-free patient samples. Moreover, there is relevant heterogeneity in pain induction methods used by the included studies. There are often differences in pain threshold and tolerance as a function of method used.^16^ Accordingly, the subgroup analyses should be interpreted with caution because it cannot be concluded from the data available here that veterans in principle have higher pain thresholds than nonveterans, but more research is needed to address this question.

### 4.3. Limitations

Several limitations of our review should be noted. First, there were a low number of studies for some of the pain perception measures. This applies in particular to CPM. A further limitation is that research on pain perception is primarily based on experimental pain tests, which somewhat lack ecological validity. Therefore, the transfer of our results to “real life” must be considered with caution because the available data were generally collected under controlled conditions.

Another important critique is the assessment of PTSD. It might be the case that those with accident-related trauma are only qualifying for subsyndromic PTSD dominated by hyperarousal syndromes, whereas victims of combat-related trauma can be more often characterized by the full symptom cluster of PTSD. In particular, the definition of the stressor criterion for PTSD (“Criterion A1”) can be discussed critically in accident-related trauma, but are clearer cut fulfilled in combat-related PTSD. Further research is warranted to investigate the relationship between different types of trauma and pain perception and to identify possible underlying mechanisms. At the same time, these results provide important information for the planning and implementation of future studies. Posttraumatic stress disorder is a very heterogeneous condition. As long as the individual studies conflate heterogeneous samples or limit themselves to individual subsamples, the relationship between PTSD and pain perception is likely to be inconsistent and the literature will be cluttered with findings in opposing directions. A greater focus on larger orchestrated studies could produce interesting findings that account more for complex relationship between PTSD and pain perception.

## 5. Conclusions

In conclusion, our data indicate that there is no clear relationship between PTSD and pain perception. However, the nature of trauma may affect pain perception differently because differential effects of PTSD on pain perception were observed. This has a central consequence for further research in this area: If alterations in pain processing not only depends of the presence of PTSD symptomatology itself, but also on the nature of the traumatic event, this would suggest that future pain treatments for patients with trauma exposure should be approached in a subgroup-specific manner.

## Disclosures

The authors have no conflicts of interest to declare.

## Appendix A. Supplemental digital content

Supplemental digital content associated with this article can be found online at http://links.lww.com/PR9/A78.

## References

[1] APA. Diagnostic and statistical manual of mental disorders. Washington: American Psychiatric Association, 2015.

[2] Bohus M Limberger M Ebner U Glocker FX Schwarz B Wernz M Lieb K Pain perception during self-reported distress and calmness in patients with borderline personality disorder and self-mutilating behavior. Psychiatry Res 2000;95:251–60. 10974364 10.1016/s0165-1781(00)00179-7

[3] Boyd J Protopopescu A O'Connor C Neufeld R Jetly R Hood H Lanius R McKinnon M Dissociative symptoms mediate the relation between PTSD symptoms and functional impairment in a sample of military members, veterans, and first responders with PTSD. Eur J Psychotraumatol 2018;17:1463794. 10.1080/20008198.2018.1463794 PMC5965037 29805778

[4] Breslau N Reboussin B Anthony J Storr C The structure of posttraumatic stress disorder: latent class analysis in 2 community samples. Arch Gen Psychiatry 2005;62:1343–51. 16330722 10.1001/archpsyc.62.12.1343

[5] Defrin R Ginzburg K Solomon Z Polad E Bloch M Govezensky M Schreiber S Quantitative testing of pain perception in subjects with PTSD—implications for the mechanism of the coexistence between PTSD and chronic pain. PAIN 2008;138:450–9. 18585862 10.1016/j.pain.2008.05.006

[6] Defrin R Lahav Y Solomon Z Dysfunctional pain modulation in torture survivors: the mediating effect of PTSD. J Pain 2017;18:1–10. 27687222 10.1016/j.jpain.2016.09.005

[7] Defrin R Schreiber S Ginzburg K Paradoxical pain perception in posttraumatic stress disorder: the unique role of anxiety and dissociation. J Pain 2015;16:961–70. 26168878 10.1016/j.jpain.2015.06.010

[8] DerSimonian R Laird N Meta-analysis in clinical trials. Control Clin Trials 1986;7:177–88. 3802833 10.1016/0197-2456(86)90046-2

[9] Diener SJ Wessa M Ridder S Lang S Diers M Steil R Flor H Enhanced stress analgesia to a cognitively demanding task in patients with posttraumatic stress disorder. J Affect Disord 2012;136:1247–51. 21733577 10.1016/j.jad.2011.06.013

[10] Dixon-Gordon K Berghoff C McDermott M Borderline personality disorder symptoms and pain in college students: the role of emotional suppression. J Pers Disord 2018;32:277–88. 28604278 10.1521/pedi_2017_31_300

[11] Dunne-Proctor RL Kenardy J Sterling M The impact of posttraumatic stress disorder on physiological arousal, disability, and sensory pain thresholds in patients with chronic whiplash. Clin J Pain 2016;32:645–53. 26560075 10.1097/AJP.0000000000000309

[12] Geisser M Roth R Bachman J Eckert T The realtionship between symptoms of post-traumatic stress disorder and pain, affective disturbance and disability among patients with accident and non-accident related pain. PAIN 1996;66:207–14. 8880842 10.1016/0304-3959(96)03038-2

[13] Geuze E Westenberg HG Jochims A de Kloet CS Bohus M Vermetten E Schmahl C Altered pain processing in veterans with posttraumatic stress disorder. Arch Gen Psychiatry 2007;64:76–85. 17199057 10.1001/archpsyc.64.1.76

[14] Ginzburg K Biran I Aryeh IG Tsur N Defrin R Pain perception and body awareness among individuals with borderline personality disorder. J Pers Disord 2018;32:618–35. 28902571 10.1521/pedi_2017_31_316

[15] Gomez-Perez L Lopez-Martinez AE Association of trauma, posttraumatic stress disorder, and experimental pain response in healthy young women. Clin J Pain 2013;29:425–34. 23183263 10.1097/AJP.0b013e31825e454e

[16] Graven-Nielsen T Vaegter H Finocchietti S Handberg G Arendt-Nielsen L Assessment of musculoskeletal pain sensitivity and temporal summation by cuff pressure algometry: a reliability study. PAIN 2015;156:2193–2202. 26172551 10.1097/j.pain.0000000000000294

[17] Green B Krupnick J Chung J Siddique J Krause E Revicki D Frank L Miranda J Impact of PTSD comorbidity on one-year outcomes in a depression trial. J Clin Psychol 2006;62:815–35. 16703602 10.1002/jclp.20279

[18] Hansen M Hyland P Armour C Andersen T Assessing the existence of dissociative PTSD in sub-acute patients of whiplash. J Trauma Dissociation 2018;16:1–16. 10.1080/15299732.2018.1451805 29547063

[19] Hartling L Hamm M Milne A Validity and inter-rater reliability testing of quality assessment instruments. Rockville: Agency for Healthcare Research and Quality (US), 2012. 22536612

[20] Harvold M MacLeod C Vaegter HB Attentional avoidance is associated with increased pain sensitivity in patients with chronic posttraumatic pain and comorbid posttraumatic stress. Clin J Pain 2018;34:22–9. 28398916 10.1097/AJP.0000000000000505

[21] Higgins J Green S Cochrane handbook for systematic reviews of interventions. The Cochrane Collaboration, 2009.

[22] Higgins J Thompson S Quantifying heterogeneity in a meta-analysis. Stat Med 2002;21:1539–58. 12111919 10.1002/sim.1186

[23] Jaramillo C Cooper D Wang C Tate D Eapen B York G Pugh M Subgroups of US Iraq and Afghanistan veterans: associations with traumatic brain injury and mental health conditions. Brain Imaging Behav 2015;9:445–55. 25963862 10.1007/s11682-015-9402-8

[24] Jenewein J Erni J Moergeli H Grillon C Schumacher S Mueller-Pfeiffer C Hassanpour K Seiler A Wittmann L Schnyder U Hasler G Altered pain perception and fear-learning deficits in subjects with posttraumatic stress disorder. J Pain 2016;17:1325–33. 27641312 10.1016/j.jpain.2016.09.002 PMC5580085

[25] Kessler R Aguilar-Gaxiola S Alonso J Benjet C Bromet E Cardoso G Degenhardt L de Girolamo G Dinolova R Ferry F Florescu S Gureje O Haro J Huang Y Karam E Kawakami N Lee S Lepine J Levinson D Navarro-Mateu F Pennell B Piazza M Posada-Villa J Scott K Stein D Ten Have M Torres Y Viana M Petukhova M Sampson N Zaslavsky A Koenen K Trauma and PTSD in the WHO world mental health surveys. Eur J Psychotraumatol 2017;8(suppl 5):1353383. 29075426 10.1080/20008198.2017.1353383 PMC5632781

[26] Kessler R Sonnega A Bromet E Hughes M Nelson C Posttraumatic stress disorder in the national comorbidity survey. Arch Gen Surv 1995;52:1048–60. 10.1001/archpsyc.1995.03950240066012 7492257

[27] Kilpatrick D Ruggiero K Acierno R Saunders B Resnick H Best C Violence and risk of PTSD, major depression, substance abuse/dependence, and comorbidity: results from the National Survey of Adolescents. J Consult Clin Psychol 2003;71:692–700. 12924674 10.1037/0022-006x.71.4.692

[28] Kraus A Esposito F Seifritz E Di Salle F Ruf M Valerius G Ludaescher P Bohus M Schmahl C Amygdala deactivation as a neural correlate of pain processing in patients with borderline personality disorder and co-occurrent posttraumatic stress disorder. Biol Psychiatry 2009;65:819–22. 19058793 10.1016/j.biopsych.2008.10.028

[29] Kraus A Geuze E Schmahl C Greffrath W Treede R-D Bohus M Vermetten E Differentiation of pain ratings in combat-related posttraumatic stress disorder. PAIN 2009;143:179–85. 19289256 10.1016/j.pain.2008.12.018

[30] Lanius R Bluhm R Lanius U Pain C A review of neuroimaging studies in PTSD: heterogeneity of response to symptom provocation. J Psychiatr Res 2006;40:709–29. 16214172 10.1016/j.jpsychires.2005.07.007

[31] Lerman I Davis BA Bertram TM Proudfoot J Hauger RL Coe CL Patel PM Baker DG Posttraumatic stress disorder influences the nociceptive and intrathecal cytokine response to a painful stimulus in combat veterans. Psychoneuroendocrinology 2016;73:99–108. 27490714 10.1016/j.psyneuen.2016.07.202

[32] Magerl W Wilk S Treede R-D Secondary hyperalgesia and perceptual wind-up following intradermal injection of capsaicin in humans. PAIN 1998;74:257–68. 9520240 10.1016/s0304-3959(97)00177-2

[33] Merskey H Bogduk N IASP task force on taxonomy. Classification of chronic pain. Seattle: IASP press, 1994 pp. 209–14.

[34] Mickleborough MJ Daniels JK Coupland NJ Kao R Williamson PC Lanius UF Hegadoren K Schore A Densmore M Stevens T Lanius RA Effects of trauma-related cues on pain processing in posttraumatic stress disorder: an fMRI investigation. J Psychiatry Neurosci 2011;36:6–14. 20964954 10.1503/jpn.080188 PMC3004970

[35] Miller M Kaloupek D Dillon A Keane T Externalizing and internalizing subtypes of combat-related PTSD: a replication and extension using the Psy-5 scales. J Abnormal Psychol 2004;113:636–45. 10.1037/0021-843X.113.4.636 15535795

[36] Moher D Liberati A Tetzlaff J Altman D; PRISMA-Group. Preferred reporting items for systematic reviews and meta-analyses: the PRISMA statement. Ann Int Med 2009;151:1–7. 21603045 PMC3090117

[37] Mostoufi S Godfrey KM Ahumada SM Hossain N Song T Wright LJ Lohr JB Afari N Pain sensitivity in posttraumatic stress disorder and other anxiety disorders: a preliminary case control study. Ann Gen Psychiatry 2014;13:31. 25422670 10.1186/s12991-014-0031-1 PMC4236800

[38] Nandi A Beard J Galea S Epidemiologic heterogeneity of common mood and anxiety disorders over the lifecourse in the general population: a systematic review. BMC Psychiatry 2009;9:31. 19486530 10.1186/1471-244X-9-31 PMC2700109

[39] Niedtfeld I Schulze L Kirsch P Herpertz S Bohus M Schmahl C Affect regulation and pain in borderline personality disorder: a possible link to the understanding of self-injury. Biol Psychiatry 2010;68:383–91. 20537612 10.1016/j.biopsych.2010.04.015

[40] Nugent N Koenen K Bradley B Heterogenity of posttraumatic stress symptoms in a highly traumatized low income, urban, African American sample. J Psychiatr Res 2012;46:1576–83. 22906539 10.1016/j.jpsychires.2012.07.012 PMC3488381

[41] Ocañez K McHugh R Otto M A meta-analytic review of the association between anxiety sensitivity and pain. Depress Anxiety 2010;27:760–7. 20336798 10.1002/da.20681

[42] Orr SP Meyerhoff JL Edwards JV Pitman RK Heart rate and blood pressure resting levels and responses to generic stressors in Vietnam veterans with posttraumatic stress disorder. J Trauma Stress 1998;11:155–64. 9479684 10.1023/A:1024421502881

[43] Pitman RK Orr SP van der Kolk BA Greenberg MS Meyerhoff JL Mougey EH Analgesia: a new dependent variable for the biological study of posttraumatic stress disorder. In: Wolf ME Mosnaim AD Wolf ME Mosnaim AD, editors. Posttraumatic stress disorder: Etiology, phenomenology, and treatment. Arlington: American Psychiatric Association, 1990 pp. 141–7.

[44] Sansone R Sansone L Borderline personality and the pain paradox. Psychiatry 2007;4:40–6. PMC2921236 20711327

[45] Schmahl C Meinzer M Zeuch A Fichter M Cebulla M Kleindienst N Ludascher P Steil R Bohus M Pain sensitivity is reduced in borderline personality disorder, but not in posttraumatic stress disorder and bulimia nervosa. World J Biol Psychiatry 2010;11:364–71. 20218798 10.3109/15622970701849952

[46] Siqveland J Hussain A Lindstrøm J Ruud T Hauff E Prevalence of posttraumatic stress disorder in persons with chronic pain: a meta-analysis. Front Psychiatry 2017;8:164. 28959216 10.3389/fpsyt.2017.00164 PMC5603802

[47] Sterling M Jull G Kenardy J Physical and psychological factors maintain long-term predictive capacity post-whiplash injury. PAIN 2006;122:102–8. 16527397 10.1016/j.pain.2006.01.014

[48] Sterling M Jull G Vicenzino B Kenardy J Darnell R Physical and psychological factors predict outcome following whiplash injury. PAIN 2005;114:141–8. 15733639 10.1016/j.pain.2004.12.005

[49] Sterling M Kenardy J The relationship between sensory and sympathetic nervous system changes and posttraumatic stress reaction following whiplash injury—a prospective study. J Psychosomat Res 2006;60:387–93. 10.1016/j.jpsychores.2005.08.016 16581363

[50] Strigo I Spadoni A Inslicht S Simmons A Repeated exposure to experimental pain differentiates combat TBI with and without PTSD. Neuropsychopharmacology 2017;43:S334–5.

[51] Strigo IA Simmons AN Matthews SC Grimes EM Allard CB Reinhardt LE Paulus MP Stein MB Neural correlates of altered pain response in women with posttraumatic stress disorder from intimate partner violence. Biol Psychiatry 2010;68:442–50. 20553750 10.1016/j.biopsych.2010.03.034

[52] Tragesser S Bruns D Disorbio J Borderline personality disorder features and pain: the mediating role of negative affect in a pain patient sample. Clin J Pain 2010;26:348–53. 20393271 10.1097/AJP.0b013e3181cd1710

[53] Vaegter HB Andersen TE Harvold M Andersen PG Graven-Nielsen T Increased pain sensitivity in accident-related chronic pain patients with comorbid posttraumatic stress. Clin J Pain 2018;34:313–21. 28799972 10.1097/AJP.0000000000000543

[54] Waelde L Silvern L Fairbank J A taxometric investigation of dissociation in Vietnam veterans. J Trauma Stress 2005;18:359–69. 16281233 10.1002/jts.20034

[55] WHO. The IDC-10 classification of mental and behavioural disorders. Diagnostic criteria for research. Geneva: World Health Organisation, 1993.

[56] Yarnitsky D Bouhassira D Drewes A Fillingim R Granot M Hansson P Landau R Marchand S Matre D Nilsen K Stubhaug A Treede R Wilder-Smith O Recommendations on practice of conditioned pain modulation (CPM) testing. Eur J Pain 2015;19:805–6. 25330039 10.1002/ejp.605

